# Immigrants, the Emergency Physician and the Election Day

**DOI:** 10.5811/westjem.2017.1.33506

**Published:** 2017-01-31

**Authors:** Bradley D. Shy

**Affiliations:** Icahn School of Medicine at Mount Sinai, Department of Emergency Medicine, New York

The most recent Election Day — extraordinary in so many ways — seemed a typical Tuesday inside the emergency department (ED) at Elmhurst Hospital Center in Queens, NY. We weren’t busy, but within hours I had treated patients from five continents. We used staff interpreters to speak to patients in Spanish and Mandarin, as well as the phone-based “language line” to converse in Russian, Bengali and Fujianese. Recent data show that 71% of residents of the Elmhurst neighborhood are foreign born, the highest proportion in New York City.1 Although our ED that day reflected this, each additional language seemed as commonplace and fitting as each new laceration, motor vehicle collision or appendicitis case.

By my next shift three days later, Donald Trump had become the president-elect and I realized how directly my patients and practice could be affected. During physical examinations, foreign-born patients nervously joked about the heightened possibility of deportation. Mindful of Trump’s campaign promise to remove three million immigrants and to defund “Sanctuary Cities” such as ours, I didn’t know how best to reassure them.^2^ I would feebly suggest that mass deportation seemed absurd or even un-American. The patients tended to smile back, polite but unconvinced.

Beyond the obvious traumatic impact on immigrants’ lives, these deportation threats would also harm the specialty of emergency medicine. The largest and most meaningful studies in emergency medicine typically include urban hospitals with significant foreign-born patient populations.^3^ More individually, physicians encounter countless immigrants and refugees over their years of training. During my own residency at Bellevue Hospital Center in New York, these patients regularly exposed their personal stories and their ailing bodies to me — often on the worst day of their lives. I would not be the doctor I am today without these people.

For emergency physicians — a politically diverse group slightly more likely to favor the Republican Party — the ironies of this immigration debate can be nauseating.^4^ Contrary to the common narrative of the presidential campaign, immigrants are significantly less likely than U.S.-born residents to come to the emergency department.^5^ Meanwhile, a 2015 Association of American Medical Colleges (AAMC) report showed how the U.S. will face a shortfall of between 61,700 and 94,700 physicians by 2025.^6^ Foreign medical graduates will be crucial to mitigating this deficit, particularly in many rural areas from which Trump drew his support.^7^ Finally, as extreme as Trump’s immigration threats may seem, his actions may only extend those of President Obama, who has deported more immigrants than any U.S. president in history.^8^

After initially submitting this article for publication, President Trump did indeed sign an executive order restricting entrance from seven predominantly Muslim nations and barring the admission of refugees from any country for 120 days. Notwithstanding the obvious danger this poses to refugees’ lives, my two hospitals will also lose. The satisfaction of providing great care to those just starting out in our country is indescribable. Beyond our patients, we may sacrifice physicians as well. With trepidation, I read about the young Sudanese doctor attempting to return to her job at the Cleveland Clinic when she was instead placed in a holding cell at John F. Kennedy Airport in New York before being eventually sent to Saudi Arabia.^9^ More broadly, Dr. Atul Grover, an executive of the AAMC, calculated that 260 individuals have applied to start their internships in the U.S. this July but may be barred as they hail from a prohibited nation.^10^ Many of my brightest colleagues are foreign born; I hope that future generations of foreign physicians still consider practicing in American hospitals.

I am in no position to predict how immigration will ultimately change under the new president, but I can and do promise this to our foreign-born patients: in the emergency room, you are welcome. This welcome extends 24 hours a day, every day, no matter the political climate outside the hospital. We will speak to you in your preferred language, provide the same care that we give to all patients, and do this without regard to your ability to pay. As we have taken oaths to do, we will never divulge your personal information to any outside entity. When your medical problem is stabilized, our social worker colleagues can help you with other concerns such as obtaining prescription drugs, legal aid, or simply a safe ride home. Speaking for my emergency nurse and physician colleagues, this is our avowed privilege — and it is the debt we owe to you.
